# Streptococcal Pyrogenic Exotoxin A-Stimulated Monocytes Mediate Regulatory T-Cell Accumulation through PD-L1 and Kynurenine

**DOI:** 10.3390/ijms20163933

**Published:** 2019-08-13

**Authors:** Katharina Giesbrecht, Sandra Förmer, Aline Sähr, Klaus Heeg, Dagmar Hildebrand

**Affiliations:** 1 Medical Microbiology and Hygiene, Centre for Infectious Diseases, University Hospital Heidelberg, 69120 Heidelberg, Germany; 2 DZIF German Center for Infection Research, 38124 Brunswick, Germany

**Keywords:** superantigen, SPEA, *Streptococcus pyogenes*, PD-L1, IDO, kynurenine, Tregs, apoptosis

## Abstract

Bacterial superantigens (SAgs) are exotoxins that promote a fulminant activation of the immune system. The subsequent intense release of inflammatory cytokines often results in hypotension, shock, and organ failure with high mortality rates. In the current paradigm, the direct and simultaneous binding of SAgs with T-cell receptor (TCR)-bearing Vβ regions and conserved structures on major histocompatibility complex class II (MHC class II) on antigen-presenting cells (APCs) induces the activation of both cell types. However, by crosslinking MHC class II molecules, APCs can be activated by SAgs independently of T lymphocytes. Recently, we showed that streptococcal pyrogenic exotoxin A (SPEA) of *Streptococcus pyogenes* stimulates an immunogenic APC phenotype with upregulated costimulatory molecules and inflammatory cytokines. Additionally, we revealed that SPEA triggers immunosuppressive programs in monocytes that facilitate the accumulation of regulatory T cells (Tregs) in in vitro monocyte/CD4^+^ T-cell cocultures. Immunosuppressive factors include anti-inflammatory interleukin 10 (IL-10), co-inhibitory surface molecule programmed cell death 1 ligand 1 (PD-L1), and the inhibitory indoleamine 2,3-dioxygenase (IDO)/kynurenine effector system. In the present study, we investigated the underlying mechanism of SPEA-stimulated monocyte-mediated accumulation of Tregs. Blood-derived monocytes from healthy donors were stimulated with SPEA for 48 h (SPEA-monocytes). For the evaluation of SPEA-monocyte-mediated modulation of CD4^+^ T lymphocytes, SPEA was removed from the culture through extensive washing of cells before adding allogeneic CD3/CD28-activated T cells. Results: In coculture with allogeneic CD4^+^ T cells, SPEA-monocytes mediate apoptosis of CD4^+^Foxp3^−^ lymphocytes and accumulation of CD4^+^Foxp3^+^ Tregs. PD-L1 and kynurenine are critically involved in the mediated cell death because blocking both factors diminished apoptosis and decreased the proportion of the CD25^+^/Foxp3^+^ Treg subpopulation significantly. Upregulation of PD-L1 and kynurenine as well as SPEA-monocyte-mediated effects on T cells depend on inflammatory IL-1β. Our study shows that monocytes activated by SPEA mediate apoptosis of CD4^+^Foxp3^−^ T effector cells through PD-L1 and kynurenine. CD4^+^Foxp3^+^ T cells are resistant to apoptosis and accumulate in SPEA-monocyte/CD4^+^ T-cell coculture.

## 1. Introduction

*Streptococcus pyogenes* (also known as Group A streptococcus (GAS)) is a Gram-positive coccus and possibly part of the microbiota of our skin and upper respiratory tract. In humans, GAS can cause a wide range of diseases [1,2]. Among those are superficial infections, such as pharyngitis and impetigo, and severe invasive infections, such as septicemia, necrotizing fasciitis, and streptococcal toxic shock syndrome (STSS) [1,2,3]. The invasiveness and pathogenesis of *Streptococcus pyogenes* strains highly depend on expressed virulence factors [4,5]. The group of streptococcal superantigens (SAgs), commonly referred to as erythrogenic toxins or streptococcal pyrogenic exotoxins, is considered as hallmark virulence factors [6]. There are more than ten genetically distinct streptococcal SAgs including the first identified SAgs, streptococcal pyrogenic exotoxin A (SPEA) and SPEC, considered to be important for severe GAS infections [7]. Indeed, it was demonstrated via a nasopharyngeal infection model that SAgs, human major histocompatibility complex class II (MHC class II) molecules, and Vβ-specific T cells are required for efficient GAS infection in mice. Here, immunization against SAgs prevented nasopharyngeal infection [8,9]. 

During infection, all bacterial SAgs, including streptococcal SAgs and *Staphylococcus aureus* (*S.aureus*) toxic shock syndrome toxin (TSST) and *S. aureus* enterotoxins [10,11], mediate an intense activation of the immune system [12,13,14]. A major hallmark of this activation is a devastating cytokine storm [15,16,17] which might lead to systemic shock. 

The SAg-mediated hyperactivation of the immune system is achieved by simultaneously binding and crosslinking MHC class II molecules on antigen-presenting cells (APCs) and T-cell receptors (TCRs) on T lymphocytes bearing susceptible Vβ regions. Thus, SAgs behave like bifunctional agents that induce polyclonal activation of up to 10% of the T-cell pool [13,18,19]. 

The initial SAg-stimulated activation of T cells that is presented by the release of cytokines such as TNFα, interleukin-2 (IL-2), and IFNγ is followed by a phase of clonal T-cell expansion which eventually results in apoptosis and clonal retraction [20,21,22,23]. The Vβ T cells that escape apoptosis (around 50%) are tolerant toward further stimulation [24]. Additionally, stimulation with SAg amplifies the CD4^+^ CD25^+^ Foxp3^+^ Treg population [25,26,27,28,29]. However, the precise mechanism leading to Treg induction is not well understood.

For T-cell activation, the presence of APCs and the binding of SAg to MHC class II molecules are necessary [24,30]. However, APCs can be activated by SAgs independently of T cells [31,32,33,34]. The interaction of MHC class II and different SAgs has been investigated. It became evident that SAgs are not only capable of binding to MHC class II but also share the ability to crosslink MHC class II molecules [31,35,36,37,38]. This suggested that SAgs might confer a signal to the APC. A study of Espel et al. revealed that direct binding of staphylococcal TSST-1 to MHC class II on human monocytes stimulated an increase of TNFα transcriptional and translational levels [34]. Palkama et al. showed that staphylococcal SEB stimulates IL-1β release in human monocytes and found a dependency on protein kinase C [32]. A further study demonstrated that staphylococcal TSST-1, SEA, and SEB upregulate IL-1β and TNFα in human monocytes and proved the involvement of increased intracellular calcium concentration, protein kinase C, and protein tyrosine kinase-mediated activation of NF-kappa B [33]. Our previously published data show the SPEA-stimulated induction of pro-inflammatory cytokines such as IL-6 and TNFα in human monocytes. This induction could be blocked by the calcineurin inhibitor cyclosporine [39]. Calcineurin is a protein phosphatase under the control of calcium and calmodulin that regulates downstream signaling. Taken together, the literature implies that SAg-mediated ligation of MHC class II increases intracellular calcium level and the activation of calcium-dependent proteins and signaling cascades that eventually induce cytokine expression.

In addition to pro-inflammatory cytokines, our published data show that SPEA triggers an immunosuppressive program in monocytes that includes the production of anti-inflammatory IL-10, the upregulation of the co-inhibitory surface molecule programmed death-ligand 1 (PD-L1), and the inhibitory indoleamine2,3-dioxygenase (IDO)/kynurenine system [39]. 

PD-L1 (also termed CD274, B7-H1) is a co-inhibitory molecule expressed by many cell types, including monocytes. Its expression is increased after infection-mediated inflammation. In detail, the activation of Toll-like receptor (TLR) signaling, the following cytokine secretion, and the subsequent cytokine receptor-mediated activation of STAT3 directly induce gene expression of PD-L1 in APCs [40]. PD-L1 binds to PD-1 (programmed cell death protein 1), a negative co-stimulator receptor on activated T cells [41,42]. The PD-l/PD-L1 binding promotes development and function of regulatory T cells (Tregs) by induction and maintenance of the Treg-specific transcription factor forkhead box protein P3 (Foxp3) [40,43]. During primary T-cell activation, PD-l/PD-L1 interaction mediates blockage of T-cell proliferation and cytokine production and inhibits cytotoxic activity and cell survival [44,45]. Additionally, effector T-cell reactivation and function is negatively modulated by the PD-1/PD-L1 interaction [46]. Altogether, PD-L1 fulfills a major role in suppressing the adaptive immune system during infection. 

IDO is also known for its role in immune suppression. The enzyme is strongly induced in APCs in response to inflammatory signals, IFNγ, interleukin 1 (IL-1), and IL-6, as well as in response to CTLA-4-mediated signaling, and it depends on signal transducer and activator of transcription 1 (STAT1) and STAT3 transcription factors [47,48]. It catabolizes the degradation of tryptophan (Trp) into derivates such as kynurenine. Although the depletion of Trp from the microenvironment itself is immunosuppressive [49], kynurenine additionally mediates immune modulatory effects. As ligand for the aryl hydrocarbon transcription (AHR) factor complex, it promotes the differentiation of activated CD4^+^ T cells into Tregs [50,51,52,53,54].

In SAg-mediated modulation of T cells, the role of PD-L1 as well as IDO-generated kynurenine has not yet been clarified. 

Our previous published data reveal that SPEA-stimulated monocytes (SPEA-monocytes) inhibit proliferation of CD3/CD28-stimulated allogeneic T lymphocytes. Furthermore SPEA-monocytes promote accumulation of Tregs [39]. In the present study, we investigated the mechanism underlying T-cell inhibition and Treg accumulation mediated by SPEA-monocytes.

## 2. Results

To investigate the influence of SPEA on antigen-presenting cells (APCs), blood-derived monocytes from healthy donors were stimulated with SPEA for 48 h (SPEA-monocytes). For the evaluation of SPEA-monocyte-mediated effects on CD4^+^ T lymphocytes, SPEA was removed from the culture after two days by washing cells three times with media. Afterward, freshly isolated allogeneic CD4^+^ T cells were added. 

**Figure 1:**
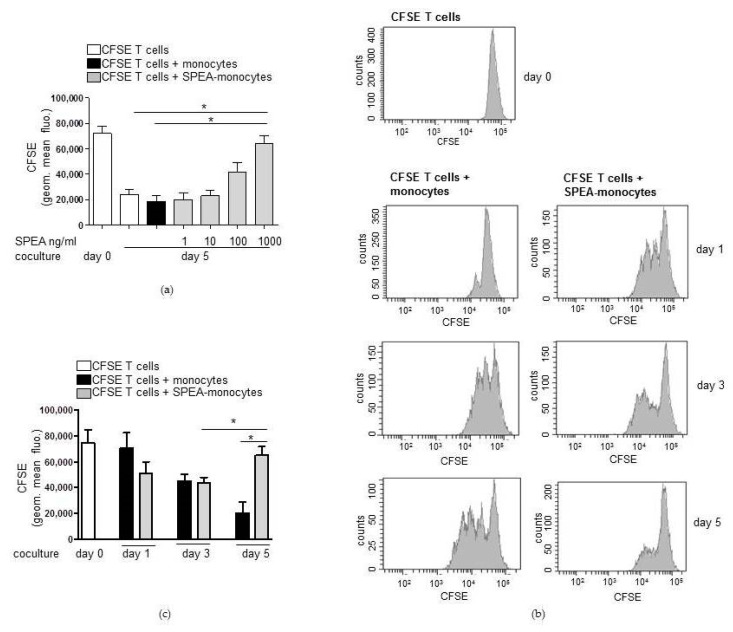
Streptococcal pyrogenic exotoxin A (SPEA)-monocytes decrease numbers of CD3/CD28-activated cocultured T cells. (**a**) CD14^+^ cells were isolated from blood, stimulated with 1 ng/mL, 10 ng/mL, 100 ng/mL, or 1000 ng/mL SPEA for 48 h (SPEA-monocytes) or left unstimulated. After washing the monocytes three times with media, cocultures with allogeneic CFSE-labeled, CD3/CD28-activated CD4^+^ T cells were started (ratio of 1:2). After five days, cell divisions of T cells were analyzed by determining the CFSE (FITC) signal using a FACSCanto. Shown is the quantification of geometric mean fluorescence of the CFSE signal (*y*-axes). The color depicts whether T cells were cultured alone (white) or together with unstimulated monocytes (black) or SPEA-monocytes (grey). (**b**) Monocytes were stimulated with 1000 ng/mL SPEA for 48 h. After washing, CFSE-T cells were added and cultured for one, three, or five days (as labeled in the graph). Depicted are FACS histograms of the CFSE (FITC) signal of T cells. (**c**) Quantification of geometric mean fluorescence of the CFSE signal of T cells at the indicated timepoints. The color depicts whether T cells were cultured alone (white) or together with unstimulated monocytes (black) or SPEA-monocytes (grey). (**a**,**c**) Columns: mean of three different donors/experiments (*n* = 3) + standard deviation (SD) as error bars. Statistical analysis: the comparison of two data groups was analyzed by Mann–Whitney U test with * *p* ≤ 0.05. The line above the columns depicts the compared groups. Kruskal–Wallis: (**a**) number of groups: 7; *p*-value 0.0187; sum value, * the medians vary significantly (*p* < 0.05). (**c**) number of groups: 7; *p*-value 0.0021; sum value, ** the medians vary significantly (*p* < 0.05).

First, we evaluated the best SPEA concentration to activate monocytes. Cells were stimulated with 1 ng/mL, 10 ng/mL, 100 ng/mL, or 1000 ng/mL SPEA for 48 h. After washing the monocytes, cells were cocultured with isolated carboxyfluorescein succinimidyl ester (CFSE)-stained and CD3/CD28-activated T cells for five days. CFSE is a cell permeable fluorescent dye covalently binding molecules intracellularly (lysine residues and other amino groups) via its succinimidyl group. During a cell division, molecules and bound CFSE are shared between daughter cells. By determining halving of the CFSE (FITC) signal at a flow cytometer, cell divisions can be analyzed. The data obtained showed that activated T cells cultured with monocytes had a decreased CFSE signal and thus proliferated, as expected. Furthermore, 1 ng/mL and 10 ng/mL SPEA-stimulated monocytes had the same stimulatory effect on T-cell proliferation. However, 100 ng/mL-stimulated APCs suppressed CD3/CD28-stimulated lowering of the CFSE signal. Thus, less divided cells were monitored. Finally, T cells cultured with 1000 ng/mL-stimulated monocytes had a significantly higher CFSE signal than activated T cells. This means that a significantly lower number of divided T cells was determined (Figure 1a). We subsequently used 1000 ng/mL SPEA to stimulate monocytes prior to T-cell coculture.

Next we performed lymphocyte growth kinetics. CFSE-labeled T cells were cocultured with SPEA-monocytes for one, three and five days and analyzed on a flow cytometer. During the first day of coculture, SPEA-monocytes seemed to increase proliferation of activated lymphocytes (Figure 1b). Nevertheless, the associated quantification of three experiments (Figure 1c) yielded no significant difference in T-cell divisions induced by monocytes and SPEA-monocytes after one and three days of culture. After five days, T-cell numbers in SPEA-monocyte coculture were significantly lower than those of unstimulated monocyte/T-cell cocultures (Figure 1b,c).

**Figure 2:**
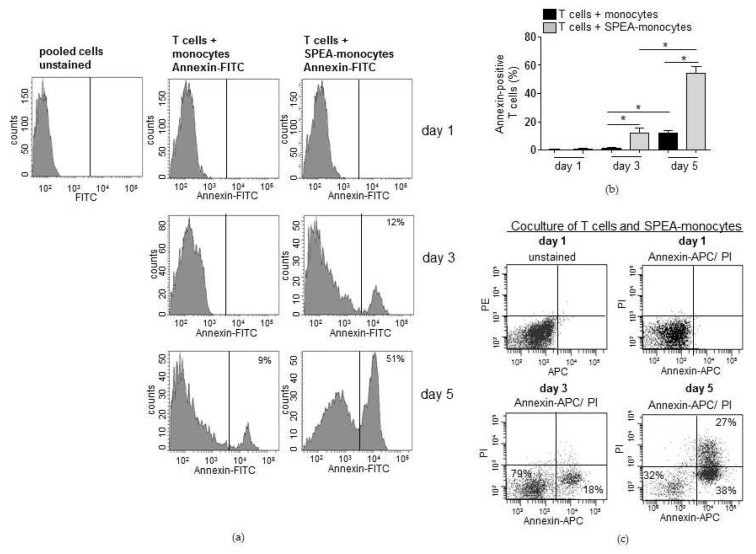
SPEA-monocytes induce apoptosis of cocultured CD4^+^ T cells. CD14^+^ cells were isolated from the blood of healthy donors, stimulated with 1000 ng/mL SPEA for 48 h (SPEA-monocytes) or left unstimulated. After washing the monocytes three times, cocultures with allogeneic CD3/CD28-activated CD4^+^ T cells were started (ratio of 1:2). After one to five days (as depicted in the graph), cells were stained with (**a**) annexin-FITC, (**b**) annexin-APC and propidiumiodide (PI), and fluorescence was analyzed using a FACSCanto. (**a**) Depicted are FACS histograms of the FITC (annexin) signal of T cells in the gated lymphocyte population. (**b**) Shown is the percentage of annexin-FITC positive T cells (*y*-axes) of data presented in (**a**) and two more experiments. Columns: mean of three different donors/experiments + SD as error bars. Black columns: T cells cocultured with unstimulated monocytes. Grey columns: T cells cocultured with SPEA-monocytes. Statistical analysis: the comparison of two data groups was analyzed by Mann–Whitney U test with * *p* ≤ 0.05. The line above the columns depicts the compared groups. Kruskal–Wallis: number of groups: 6; *p*-value 0.0114; sum value, * the medians vary significantly (*p* < 0.05). (**c**) Depicted are FACS dot plots of annexin-APC (*x*-axis) and propidiumiodide (PI, *y*-axis) double-stained cells and unstained cells, gated lymphocyte population. Day 1, day 3, and day 5 stand for the duration of SPEA-monocyte/T-cell coculture.

As the analyzed CFSE signal conveys that many divided T cells disappeared after five days of SPEA-monocyte coculture, apoptosis of lymphocytes was analyzed. We performed annexin V staining to detect cell surface changes associated with early apoptosis events. Annexin-positive (apoptotic) T cells in the SPEA-monocyte coculture remarkably increased from three (around 10%) to five (around 50%) days. Significantly fewer (around 10% at day five) lymphocytes cultured with unstimulated monocytes were apoptotic (Figure 2a,b). To further strengthen that result, we stained the SPEA-monocyte/T-cell coculture after five days with propidiumiodide (PI) in addition to annexin V. PI is a fluorescent membrane impermeable DNA intercalating agent that stains only necrotic, dead cells. Analysis of the gated double-stained lymphocytes confirmed the increase of apoptotic T cells during coculture. From day 3 to day 5 annexin-positive T cells increased PE (PI) signal and therefore became necrotic (Figure 2c).

**Figure 3:**
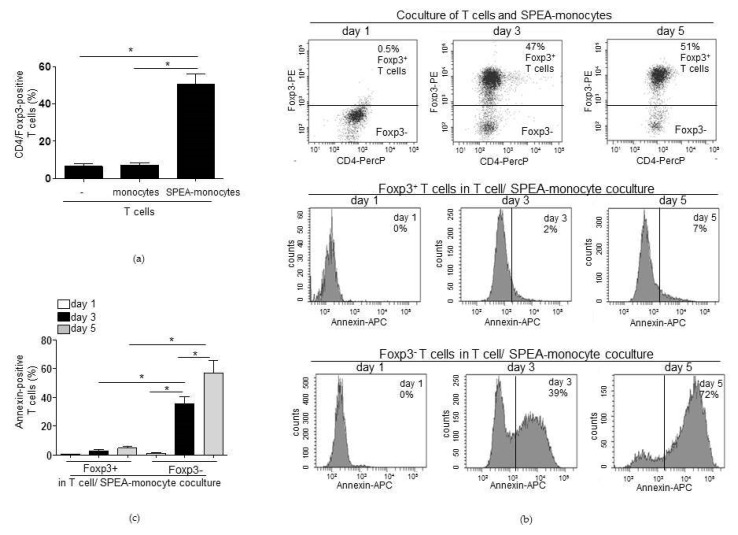
SPEA-monocytes induce apoptosis of Foxp3-negative T cells. (**a**) CD4^+^ T cells were cultured with unstimulated monocytes or SPEA-monocytes for five days. Cells were stained with anti-CD4-PercP and anti-Foxp3-PE and analyzed for double-positive cells on a FACSCanto. *Y*-axes: Percentage of CD4/Foxp3-positive Tregs. (**b**) CD4^+^ T cells were cocultured with allogeneic SPEA-monocytes for the indicated timepoints (one, three, or five days). Cells were stained with anti-CD4-PercP, anti-Foxp3-PE, and annexin-APC and analyzed on a FACSCanto. After gating for Foxp3^+^ and Foxp3^−^ cells, the fluorescent annexin signal was detected in both populations. Upper row: dot blot of Foxp3-PE/CD4-PercP double stain. Middle row: histogram of annexin-APC signal on gated Foxp3^+^ cells. Lower row: histogram of annexin-APC signal on gated Foxp3^−^ cells. (**c**) Shown is the percentage of annexin-positive T cells from (**b**) and two more experiments. White column: day 1. Black columns: day 3. Grey columns: day 5. (**a**,**c**): Columns: mean of three different donors/experiments (*n* = 3) + SD as error bars. Statistical analysis: the comparison of two data groups was analyzed by Mann–Whitney U test with * *p* ≤ 0.05. Line above the columns: compared groups. Kruskal–Wallis: (**a**) number of groups: 3; *p*-value 0.0201; sum value, *; (**b**) number of groups: 6; *p*-value 0.0099; sum value, **, the medians of (**a**,**b**) vary significantly (*p* < 0.05).

This raised the question whether the previously observed accumulation of Tregs in SPEA-monocyte coculture [39] could be due to a higher resistance of Tregs toward apoptosis. At first, we confirmed the accumulation of Tregs in cocultures of SPEA-monocytes/CD4^+^ T cells (Figure 3a). After five days, no difference in Treg numbers of cultured T cells and monocyte cocultured T cells was found, as expected. However, in T-cell/SPEA-monocyte coculture a significant increase in Tregs could be observed (Figure 3a). 

Next, cells of the SPEA-monocyte/T-cell coculture were stained with annexin, anti-Foxp3 antibody, and anti-CD4 antibody. The staining with anti-Foxp3 antibody enabled discrimination between Tregs and non-Tregs at the flow cytometer. The data again confirmed the accumulation of Foxp3-positive lymphocytes after five days of cocultures (Figure 3b, upper row: dot blot of Foxp3-PE/CD4-PercP double stain). We gated on Foxp3-positive or Foxp3-negative T cells that are both present in the mixed CD4-positive T-cell population and quantified the annexin signal of both populations (Figure 3c). After three days, around 40% of Foxp3-negative CD4^+^ T cells bound annexin, and two days later around 70% were annexin-positive (Figure 3b,c). In contrast to Foxp3^−^ cells, very few Foxp3^+^ T cells bound annexin (day 3 around 2%, day 5 around 7 %, Figure 3b,c).

**Figure 4:**
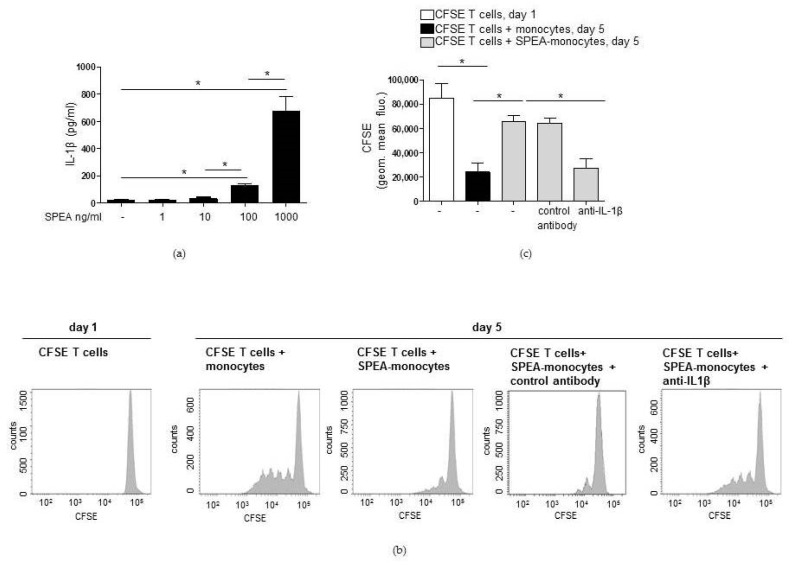
IL-1β modulates SPEA-monocytes toward a T-cell suppressing phenotype. (**a**) Primary monocytes were stimulated with 1 ng/mL, 10 ng/mL, 100 ng/mL, or 1000 ng/mL SPEA for 48 h. Supernatant was collected and used for IL-1β ELISA analyses. Columns represent the mean of three experiments + standard deviation (error bars). (**b**) Monocytes were stimulated with 1000 ng/mL SPEA for 48 h (SPEA-monocytes) or left unstimulated (monocytes). Additionally, cells were treated with 3 µg/mL anti-IL1β neutralizing antibody (anti-IL1β) or 3 µg/mL of the respective isotope control (anti-IL1β). After two days, cells were washed and allogeneic CFSE-labeled CD4 T cells (ratio 1:2) were added. After five days, the CFSE (FITC) signal was analyzed on a FACSCanto. Shown are flow cytometer histograms of the CFSE (FITC) signal of T cells. (**c**) Quantification of (**b**) and two more experiments. The color of the columns shows whether the T cells were cultured alone for a day (white) or with unstimulated monocytes (black) or SPEA-monocytes (grey) for five days. Mean + standard deviation of *n* = 3. Statistical analysis: the comparison of two data groups was analyzed by Mann–Whitney U test with * *p* ≤ 0.05. The line above the columns depicts the compared groups. Kruskal–Wallis: (**a**) number of groups: 5; *p*-value 0.0199; sum value, *; (**c**) number of groups: 5; *p*-value 0.0042; sum value, **, the medians of (**a**,**c**) vary significantly (*p* < 0.05).

Next, we aimed to clarify the mechanisms responsible for apoptosis in Foxp3^−^ T cells. From our former studies, it is known that SPEA-monocytes release high amounts of pro-inflammatory cytokines, upregulate T-cell co-inhibitory PD-L1 on their surface, and generate immunosuppressive kynurenine [39]. Immunosuppressive myeloid cells are known to inhibit T effector cells via PD-L1 and kynurenine [54]. After stimulation with bacterial components, TLR-signaling-induced IL-1β is responsible for the induction of both factors and the reprogramming of APCs toward an immunosuppressive phenotype that inhibits T-cell activation [54]. To test whether IL-1β could also play a role in the SPEA-monocyte-mediated effects, we checked the concentration of IL-1β after SPEA stimulation. We observed that 1 ng/mL and 10 ng/mL SPEA did not induce a noteworthy release of IL-1β. However, 100 ng/mL SPEA induced a significant increase in cytokine expression, and 1000 ng/mL SPEA-stimulated monocytes released the highest amount of IL-1β and significant more cytokine than unstimulated and 100 ng/mL SPEA-stimulated cells (Figure 4a). Then, we blocked IL-1β-signaling. During SPEA stimulation, monocytes were treated with anti-IL-1β neutralizing antibody. After two days, CFSE-labeled CD4^+^ T cells were added to washed monocytes for five days. To exclude any unspecific effect of the antibody, we included the anti-IL1β isotype antibody in the experiments. T cells cultured for five days with untreated monocytes proliferated, as expected. In SPEA-monocyte coculture, very few divided lymphocytes were detected. Treatment with the isotype antibody had no effect on T-cell proliferation. However, blocking IL-1β-signaling decreased the CFSE signal of SPEA-monocyte cocultured T cells significantly (Figure 4b,c). This result implies an impact of IL-1β-signaling in the SPEA-monocyte-mediated effect on T-cell activation.

**Figure 5:**
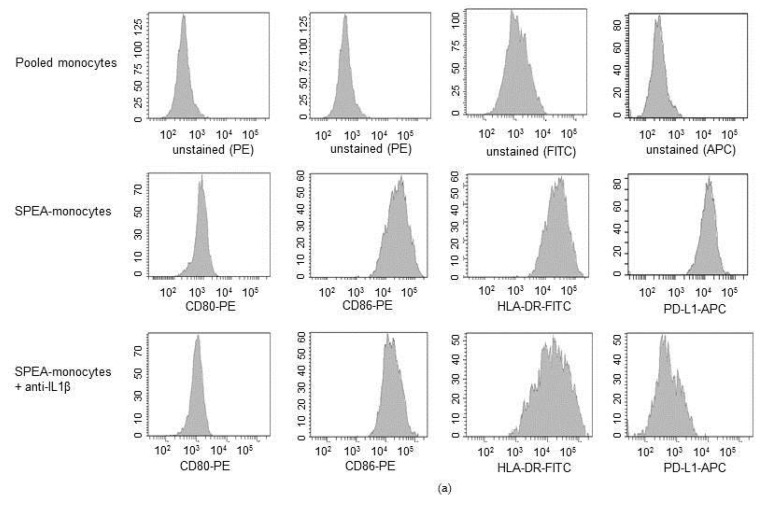

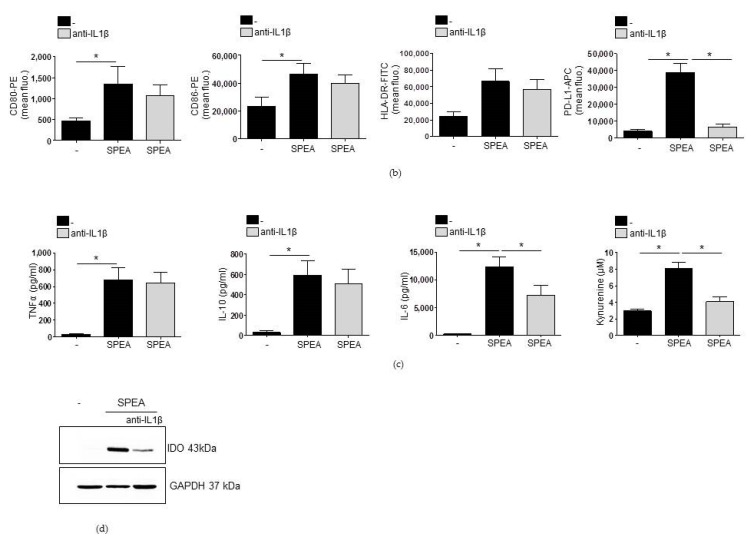
SPEA-stimulated induction of PD-L1 and kynurenine depends on IL-1β. Primary monocytes were stimulated with 1000 ng/mL SPEA +/− anti-IL-1β neutralizing antibody (3 µg/mL) for 48 h. Antibody staining was performed with anti-CD80-PE, anti-CD86-PE, anti-HLA-DR-FITC and anti-PD-L1-APC antibodies. Fluorescence was analyzed on a FACSCanto. (**a**) Histograms of antibody staining. Upper row: fluorescence signal of unstained, pooled monocytes. Middle row: data of SPEA-monocytes. Bottom row: data of SPEA-monocytes + anti-IL-1β. (**b**) Quantification of mean fluorescence of (**a**) and two more experiments. (**c**) Supernatant of cells was used for ELISA analyses. (**d**) Western blot analysis of IDO and GAPDH (loading control) of SPEA-stimulated monocytes (48 h) +/− anti-IL-1β neutralizing antibody. (**b**,**c**): Black columns: culture without anti-IL1β antibody. Grey columns: with supplemented anti-Il-1β. Shown is the mean + SD, *n* = 3. The comparison of two data groups was analyzed by Mann–Whitney U test with * *p* ≤ 0.05. The lines above the columns depict the compared groups. Kruskal–Wallis for (**b**,**c**): number of groups: 3; CD80, CD86, HLA-DR: *p*-value 0.0509; sum value, n.s. TNF, IL-10: *p*-value 0.0608; sum value (CD80, CD86, HLA-DR, TNF, IL-10), n.s. PD-L1, kynurenine: *p*-value 0.0147. IL-6: *p*-value 0.0390, sum value (PD-L1, kynurenine, IL-6), * the medians vary significantly (*p* < 0.05).

Then, we investigated the link between SPEA-stimulated IL-1β and SPEA-monocyte-mediated effects on T cells. We evaluated how blocking IL-1β during SPEA-stimulation modulates the previously demonstrated SPEA-monocyte phenotype, including upregulation of costimulatory CD80, CD86, inhibitory PD-L1, inflammatory TNFα, IL-6 and anti-inflammatory IL-10, and kynurenine [39]. Flow cytometry data (Figure 5a,b) confirmed that 1000 ng/mL SPEA stimulated upregulation of CD80, CD86, HLA-DR (human MHC class II), and PD-L1. SPEA-stimulated induction of costimulatory CD80, CD86, and HLA-Dr surface expression was not significantly modulated after anti-IL-1β antibody treatment. However, PD-L1 expression was significantly repressed by neutralizing IL-1β (Figure 5a,b).

Additional ELISA analysis confirmed that 1000 ng/mL SPEA induced the production of TNFα, IL-6, and IL-10 (Figure 5c). TNFα and IL-10 release was not changed significantly after blocking the IL-1 receptor. However, IL-6 production was reduced significantly. Furthermore, the generation of SPEA-induced kynurenine, the product of the IDO-catalyzed degradation of tryptophan, was potently and significantly inhibited through IL-1β-neutralization (Figure 5c). As expected from the decreased kynurenine generation, SPEA-stimulated IDO expression, shown by Western blot analysis after 48 h of stimulation, was also decreased in monocytes after treatment with anti-IL1β antibody (Figure 5d).

**Figure 6:**
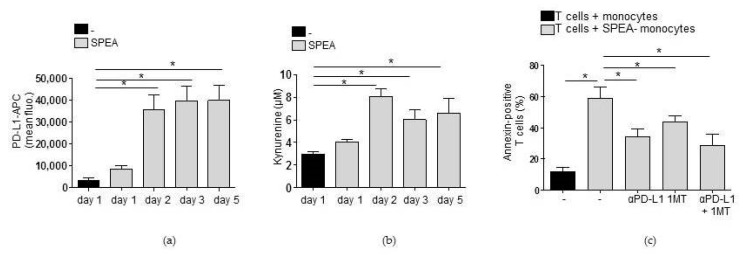
Apoptosis of T cells in SPEA-monocyte/T-cell coculture is mediated by PD-L1 and IDO. (**a**,**b**) Primary monocytes were stimulated with 1000 ng/mL SPEA untreated. After 48 h, cells were washed and T cells were added. On days 1 and 2 (before coculture) and on days 3 and 5, cells were analyzed. (**a**) Antibody staining was performed with anti-PD-L1-APC antibody. Fluorescence was analyzed on a FACSCanto. Shown is the mean fluorescence of the APC signal of the gated monocyte population. (**b**) Supernatant of cultures was used for the detection of kynurenine. (**c**) Monocytes were stimulated with 1000 ng/mL SPEA for 48 h or left unstimulated. Additionally, cells were treated with the IDO inhibitor 1MT (240 µM, 1 h prior to SPEA-stim.), anti-PD-L1 antibody (10 µg/mL, after the start of coculture) or both as indicated in the graph. After two days, cells were washed, anti-PD-L1 antibody was supplemented, and allogeneic CFSE-labeled CD4^+^ T cells (ratio 1:2) were added. After five days, cells were stained with annexin-FITC and the gated lymphocyte population was analyzed on a FACSCanto. Shown is the percentage of annexin-positive (apoptotic) T cells. (**c**) Black columns: T cells cultured with unstimulated monocytes. Grey columns: T cells cultured with SPEA-monocytes, treated as indicated. Columns depict the mean + SD, *n* = 3. (**a**,**b**,**c**) Statistical analysis: the comparison of two data groups was analyzed by Mann–Whitney U test with * *p* ≤ 0.05. Kruskal–Wallis: (**a**) number of groups: 5; *p*-value 0.0255; sum value, * the medians vary significantly (*p* < 0.05); (**b**) number of groups: 5; *p*-value 0.0241; sum value, * the medians vary significantly (*p* < 0.05); (**c**) number of groups: 5; *p*-value 0.0166; sum value, * the medians vary significantly (*p* < 0.05).

These results educed the hypothesis that neutralization of IL-1β in SPEA-monocytes reversed T-cell inhibition through modulation of monocyte phenotype and abrogation of PD-L1 and IDO-generated kynurenine. To review this hypothesis, we first investigated whether PD-L1 surface expression and kynurenine concentration in the cell supernatant were stable during the coculture of monocytes and T cells. We stimulated monocytes with SPEA and analyzed PD-L1 surface expression via flow cytometry (Figure 6a) and kynurenine concentration (Figure 6b) after one and two days. Then, cells were washed and T cells were added as described before. Three and five days later, PD-L1 expression was analyzed on a flow cytometer in the gated monocyte population. Kynurenine concentration in the coculture was determined. PD-L1 expression (Figure 6a) remained stable, and kynurenine was still produced after the start of coculture (Figure 6b). Then, we blocked the generation of kynurenine via a competitive IDO inhibitor (1MT) prior to coculture and prevented PD-1/PD-1 binding in SPEA-monocyte/CD4^+^ T-cell coculture by an anti-PD-L1 antibody. Afterward, apoptosis of T cells was determined by analyzing annexin staining. SPEA-monocytes mediated apoptosis of lymphocytes, as expected (Figure 6c). Blocking PD-1 signaling in T cells induced a significant decrease in annexin binding and thereby an increase in survival of SPEA-monocyte cocultured T cells. Inhibition of kynurenine generation (by 1MT) resulted in a similar decrease of apoptosis. Treatment with 1MT and anti-PD-L1 did not further increase survival significantly.

**Figure 7:**
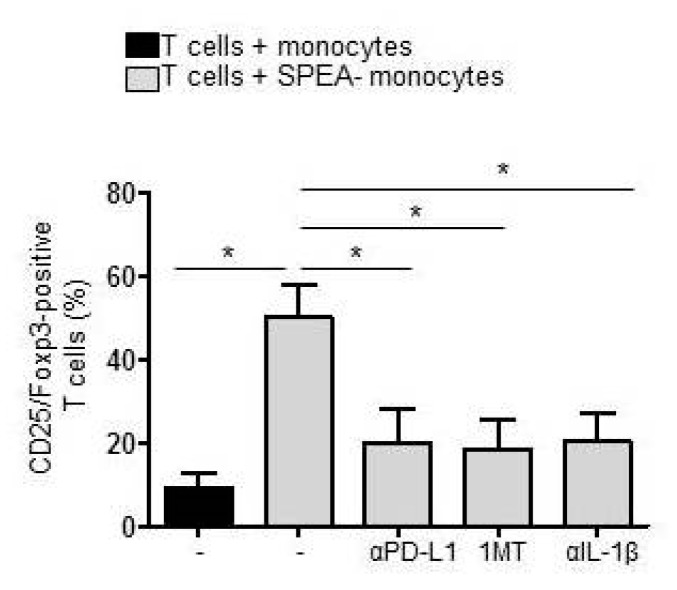
Accumulation of Tregs in SPEA-monocyte/T-cell coculture depends on PD-L1 and IDO. Monocytes were stimulated with SPEA for 48 h or left unstimulated. Additionally, cells were treated with 1MT (240 µM, 1 h prior to SPEA stimulation) or anti-IL-1β antibody (3 µg/mL during stimulation). After two days, cells were washed, anti-PD-L1 antibody (10 µg/mL) was supplemented when indicated in the graph, and allogeneic CD3/CD28-activated CD4 T cells (ratio 1:2) were added. After five days, cells were stained with fluorescent anti-CD25 and anti-Foxp3 antibodies and analyzed on a FACSCanto. Shown is the percentage of CD25/Foxp3-positive T cells in the T-cell population. Black columns: T cells cultured with unstimulated monocytes. Grey columns: T cells cultured with SPEA-monocytes, treated as indicated. Columns depict the mean + SD (error bars) of four experiments. Statistical analysis: Mann–Whitney U test with * *p* ≤ 0.05. The line above the columns depicts the compared groups. Kruskal–Wallis: number of groups: 5; *p*-value 0.0130; sum value, * the medians vary significantly (*p* < 0.05).

Finally, the accumulation of Tregs was analyzed after five days of coculture. SPEA-monocytes mediated the accumulation of Tregs (38–55%), as expected (Figure 7). Administration of anti-PD-L1 antibody diminished the number of CD25/Foxp3^+^ Tregs significantly (14–28%), just as inhibition of kynurenine generation via 1MT (12–24%). Consequently, treatment with the anti-IL-1β antibody during monocyte stimulation that suppressed expression of PD-L1, expression of IDO, and generation of kynurenine also potently inhibited Treg accumulation (Figure 7).

## 3. Discussion

A direct and simultaneous binding of SAg with T-cell receptors (TCRs) and MHC class II on APCs is the current accepted paradigm for T-cell activation by SAgs. In this scenario, SAgs must reach a site in the body with both cell types. During infection, the draining lymph node is the most likely site for SAg to interact with Vβ T cells as well as APCs. The idea of SAgs binding T cells in peripheral tissues or in the blood is rather unlikely due to the limited numbers of T cells. More likely, SAgs can reach the lymph node near the infection as soluble antigen in lymphatic fluid or presented on APCs that previously inserted the SAg in the tissue at the site of infection [55]. APCs are present in all tissues and migrate after activation through an antigen to the next lymph node to encounter circulating T cells. Therefore, it seems feasible that SAgs in tissue or blood activate APCs to mediate inflammation, to migrate to the nearby lymph node and activate T cells without the need of Sag–TCR binding. Several studies show that SAgs activate APCs, independently of T cells [31,32,33,39]. SAg binding and crosslinking of MHC class II delivers a signal to the APC that accounts for the intracellular increase of calcium and eventually leads to the upregulation of pro-inflammatory cytokines. Thus, Hopkins et al. showed that in the human system SAg-induced ligation of MHC class II on monocytes upregulates TLR4 and enhanced pro-inflammatory responses to TLR ligands, independently of T cells and INFγ [56]. Other studies reveal that SAg-stimulated APCs release pro-inflammatory cytokines and upregulate costimulatory surface molecules in the absence of a TLR ligand [31,32,33,34,57]. Previously, we observed an induction of the immunosuppressive factors PD-L1 and kynurenine after SPEA stimulation of monocytes [39]. This finding let us hypothesize that activating as well as inactivating effects on T cells could be mediated by SAg-stimulated APCs. 

In the present study, we showed that SPEA-monocytes induce apoptosis of Foxp3-negative lymphocytes and accumulation of Tregs through PD-L1 and kynurenine. In our approach, we washed SPEA-stimulated monocytes several times before adding allogeneic CD4^+^ T cells. We cannot exclude the possibility that potentially ingested SPEA was presented to T cells and that a direct binding of monocytes and T cells occurred in coculture. However, stimulation of monocytes with SPEA highly upregulated PD-L1 and kynurenine. Inhibition of both factors diminished the mediated apoptosis of cocultured T cells significantly. 

Recently, we showed that TLR-activated immunogenic APCs reprogram themselves toward an immunosuppressive phenotype with high surface expression of PD-L1 and high release of kynurenine. As a trigger for this remodeling, we identified IL-1β that augments IL-6 production and a STAT3-dependent induction of immunosuppressive factors [54]. SPEA-stimulated monocytes seem to undergo a similar remodeling, as neutralization of the cytokine during SPEA stimulation diminished PD-L1 surface expression and kynurenine release. Additionally, preventing PD-L1 binding to PD-1 on T cells as well as suppression of Trp degradation into kynurenine diminished SPEA-monocyte-mediated apoptosis of effector T cells and decreased the shift of T-cell populations toward Tregs.

It is well known that the simultaneous binding of SAgs of MCH class II and TCR results in apoptosis of roughly 50% of the initial numbers of Vβ-bearing T cells after the initial activation of a phase of clonal T-cell expansion [21,22,23,58]. The surviving cells show an anergic phenotype [24,59]. Additionally, the proportion of CD4^+^ CD25^+^ Foxp3^+^ Tregs is strongly augmented [25,26,27]. The underlying mechanism of T-cell apoptosis is not entirely clarified. Several reports have suggested that Fas governs T-cell apoptosis after repeated administration of SAg [60,61,62]. Several other studies have shown that after a single dose of Sag, T cells died in the absence of Fas or Fas ligand [63,64,65,66,67]. The second proposed SAg-mediated way of T-cell death is the regulation of Bcl-2 family members [64]. Bcl-2 is the prototype member of a large family of related proteins having pro- or anti-apoptotic function by regulation of the mitochondria pathway of apoptosis [68,69]. At the end of a T-cell response, before cells die in vivo, the majority of the activated T cells exhibit decreased levels of pro-survival Bcl-2 [70]. Interestingly, SEB-induced apoptosis of T cells can be prevented by retroviral restoration of Bcl-2 [64].

According to the data of the present study, PD-L1 and kynurenine are critically involved in SPEA-monocyte-mediated T-cell apoptosis. From the literature, it is known that PD-L1/PD-1 binding induces signaling events that reduce effector T-cell survival. Inhibiting PI3K activation through the recruitment of phosphatases PD-1 suppresses CD28-mediated induction of the pro-survival Bcl-2 family member Bcl-xL [71]. Additionally, a publication on HIV infection illustrates a kynurenine-dependent mechanism through IL-2 signaling for reduced CD4^+^ T-cell survival that involves reactive oxygen species [72]. 

While effector T cells depend on pro-survival Bcl-2 member expression [73], Bcl-2 and Bcl-xL are dispensable for Treg survival [73,74,75]. Our experiments imply that Tregs overcome PD-L1- and kynurenine-mediated apoptosis. However, the involved signaling cascades in T lymphocytes must be investigated in future studies. 

In summary, we show that SPEA-stimulated monocytes induce apoptosis of Foxp3^−^ lymphocytes through PD-L1 and kynurenine. Foxp3^+^ lymphocytes are resistant to apoptosis and accumulate. The question of whether the mechanism presented here applies to other SAgs, such as the enterotoxins of *S. aureus*, cannot be answered by our work and should be clarified in future studies.

## 4. Materials and Methods 

Reagents: Streptococcal pyrogenic exotoxin A (SPEA) was purchased from Toxin Technology Inc. (Sarasota, FL, USA), IDO inhibitor 1-methyl-tryptophan (1MT) from Sigma-Aldrich (Taufkirchen, Germany), α-IL-1β and α-PD-L1 antibodies from eBioscience (Frankfurt, Germany).

Isolation/culture of CD14^+^ monocytes and CD4^+^ T cells: PBMCs were isolated from fresh blood from healthy donors by density gradient centrifugation (Biocoll separating solution, 1.077 g/mL, Biochrom AG, Berlin, Germany). CD14^+^ cells (or CD4^+^) were magnetically labeled with beads (Miltenyi Biotec, Bergisch Gladbach, Germany; Monocyte Isolation KIT 2 human, CD4^+^ T Cell Isolation KIT human) and selected via the autoMACS separator (autoMACS, program: possel, Miltenyi Biotec, Bergisch Gladbach, Germany) according to the manufacturer’s protocol. Purified monocytes (1 × 10^6^ cells/mL) were cultured in RPMI 1640 (Sigma-Aldrich, Taufkirchen, Germany) supplemented with 100 IU/mL penicillin, 100 μg/mL streptomycin, and 10% heat inactivated fetal calf serum (FCS) (Promocell, Heidelberg, Germany) at 37 °C in a humidified atmosphere in the presence of 5% CO_2_. In coculture, SPEA-stimulated and unstimulated monocytes were washed three times with RPMI after 48 h. Then, freshly isolated allogeneic CD4^+^ T cells were added. When indicated, T cells were activated with anti-CD3- and anti-CD28-coated beads (T Cell Activation/Expansion Kit, Miltenyi Biotec, Bergisch Gladbach, Germany) at a ratio of 1:2 during the assay. Format: 96-well plates. In 200 µL RPMI (plus 100 IU/mL of penicillin, 100 μg/mL streptomycin, and 10% heat inactivated fetal calf serum), 100,000 monocytes and 200,000 T cells. 

Treatment of cells: Monocytes were stimulated with 1 ng/mL, 10 ng/mL, 100 ng/mL, or 1000 ng/mL SPEA for 48 h. For inhibitor experiments, cells were treated with 240 µM 1MT (1 h prior to SPEA stimulation of monocytes and after the start of monocyte/T-cell coculture), 10 µg/mL anti-PD-L1 (after the start of coculture), 3 µg/mL α-IL-1β (parallel to SPEA-stimulation of monocytes). 

CFSE-proliferation assay: Monocytes were stimulated with SPEA in the indicated concentration. After two days, monocytes were washed three times with 1 mL RPMI medium. Then, monocytes were cultured together with freshly isolated allogeneic, CFSE-labeled, CD3/CD28-activated CD4^+^ T cells in a ratio of 1:2 in RPMI (plus 100 IU/mL of penicillin, 100 μg/mL streptomycin, and 10% heat inactivated fetal calf serum). Cells were cultured in 96-well plates. In 200 µL RPMI, 100,000 monocytes and 200,000 T cells. For CFSE labeling (before coculture), CD4^+^ T cells were incubated 10 min at RT in 0.3 mM CFSE/PBS (Molecular Probes, San Diego, CA, USA) and thereafter intensively washed with RPMI and 10% FCS. When indicated, T cells were activated with anti-CD3- and anti-CD28-coated beads (T Cell Activation/Expansion Kit, Miltenyi Biotec, Bergisch Gladbach, Germany) at a ratio of 1:2 during the assay. After one to five days of coculture, cell divisions were analyzed by determining the FITC signal using a FACSCanto (BD Biosciences, San Jose, CA, USA).

Flow cytometry: 48 h after SPEA stimulation, monocytes were analyzed for surface markers with antibody staining (1 h on ice): α-HLA-DR-FITC, α-CD80-PE, α-CD86-PE, α-CD4-PerCp (BD Biosciences, Heidelberg, Germany), and α-PD-L1-APC (eBioscience, Frankfurt, Germany). Foxp3 expression in T cells was assessed using an anti-human Foxp3 Staining Kit (e-Biosciences Frankfurt, Germany, San Diego, CA, USA). For the detection of apoptosis, annexin V APC, or annexin V Fitc (Thermo Fisher Scientific, Karlsruhe, Germany), was added (5 µL to 100 µL PBS) 10 min prior to measurement. For viability stain, a 2 µL portion of propidiumiodide (Carl Roth, Karlsruhe, Germany) was added to 100 µL PBS in addition to annexin V. Mean fluorescence (mean fluo.) was recorded using the FACS DIVA V 4.12 software on a FACSCanto (BD Biosciences, San Jose, CA, USA). 

Enzyme-linked immunosorbent assay (ELISA): Commercially available ELISA kits were used for the detection of human IL-10, IL-6, and TNFα (BD OptEIA ELISA Set; BD Biosciences Pharmingen, Heidelberg, Germany). Assays were performed with cell-free supernatants according to the manufacturer’s instructions. Absorbance was measured on a SUNRISE Absorbance reader (Tecan, Salzburg, Austria) and analyzed with Magellan V 5.0 software (Tecan, Salzburg, Austria).

Kynurenine detection: Cell culture supernatant (150 µL) was supplemented with 50 µL trichloric acid (30%) for protein precipitation and incubated at 50 °C for 30 min. After centrifugation, 75 µL of supernatant were transferred into 96-well plate format and supplemented with 75 µL Ehrlich reagent (Sigma-Aldrich, Darmstadt, Germany). For the measurement of kynurenine, a respective standard was used with the highest concentration of 50 µM, diluted stepwise 1:2. After 5 min, absorbance was measured at 492 nm with a reference wavelength of 690 nm using a photometer (SUNRISE Absorbance reader, Tecan, Salzburg, Austria). Kynurenine concentrations were calculated with the Magellan V 5.0 software (Tecan, Salzburg, Austria). 

Western blotting: 2 × 10^6^ cells were harvested and washed with PBS. For whole cell lysates, monocytes were lysed in 50 µL RIPA buffer (50 mM Tris-HCl, pH7.4; 1% Igepal; 0.25% sodium deoxycholate; 150 mM NaCl; 1 mM EDTA; 1 mM PMSF; 1 mg/mL each aprotinin, leupeptin, and pepstatin; 1mM Na3VO4; and 1 mM NaF). Samples were vortexed and incubated 30 min on ice. Lysates were then cleared via centrifugation at 14,000× *g* for 20 min. Equal amounts of cell lysates were used for separation by SDS-PAGE (12.5%). After semi-dry transfer onto nitrocellulose membranes (Whatman Protran nitrocellulose membrane; neoLab, Heidelberg, Germany), the latter were blocked with 5% BSA in TBS/0.1% Tween-20 for 1 h at RT. Probing was performed with antibodies: anti-IDO antibody and anti-GAPDH antibody (1:1000 in TBS/0.1% Tween-20, overnight) from Cell Signaling Technology (Danvers, MA, USA) and the respective secondary antibodies (1:5000 in TBS/0.1% Tween-20, 1 h) from the same company. Detection was based on enhanced chemiluminescence (ECL; PerkinElmer, Groningen, Netherlands).

Statistical analysis: The comparison of two data groups was analyzed by Mann–Whitney U test (one-tailed, confidence intervals 95%) with * *p* ≤ 0.05. Additionally, Kruskal–Wallis Test (one-way ANOVA on ranks) was performed. Software: GraphPad Prism Version 5.0 (GraphPad Software Inc., San Diego, CA, USA).

Ethical statement: This study (taking of blood samples from healthy donors and treatment of blood leukocytes with microbial stimuli) was carried out in accordance with the recommendations of the ethics committee of the Medizinische Fakultät Heidelberg with written informed consent from all subjects. All subjects gave written informed consent in accordance with the Declaration of Helsinki. The animal experiments were approved by the governmental animal ethics committee (Regierungspraesidium Karlsruhe, file number: 35-9185.81/G-132/15) and conducted according to international FELASA recommendations. The study was reviewed and approved by the ethics committee of Medizinische Fakultät Heidelberg.

## References

[1] Carapetis J.R., Steer A.C., Mulholland E.K., Weber M. (2005). The global burden of group A streptococcal diseases. Lancet Infect. Dis..

[2] Parks T., Smeesters P.R., Steer A.C. (2012). Streptococcal skin infection and rheumatic heart disease. Curr. Opin. Infect. Dis..

[3] Lappin E., Ferguson A.J. (2009). Gram-positive toxic shock syndromes. Lancet Infect. Dis..

[4] Smeesters P.R., Dreze P.A., Perez-Morga D., Biarent D., Van Melderen L., Vergison A. (2010). Group A streptococcus virulence and host factors in two toddlers with rheumatic fever following toxic shock syndrome. Int. J. Infect. Dis..

[5] Johansson L., Thulin P., Low D.E., Norrby-Teglund A. (2010). Getting under the skin: The immunopathogenesis of streptococcus pyogenes deep tissue infections. Clin. Infect. Dis..

[6] McCormick J.K., Yarwood J.M., Schlievert P.M. (2001). Toxic shock syndrome and bacterial superantigens: An update. Annu. Rev. Microbiol..

[7] Commons R.J., Smeesters P.R., Proft T., Fraser J.D., Robins-Browne R., Curtis N. (2014). Streptococcal superantigens: Categorization and clinical associations. Trends Mol. Med..

[8] Kasper K.J., Zeppa J.J., Wakabayashi A.T., Xu S.X., Mazzuca D.M., Welch I., Baroja M.L., Kotb M., Cairns E., Cleary P.P. (2014). Bacterial superantigens promote acute nasopharyngeal infection by streptococcus pyogenes in a human mhc class ii-dependent manner. PLoS Pathog..

[9] Zeppa J.J., Kasper K.J., Mohorovic I., Mazzuca D.M., Haeryfar S.M.M., McCormick J.K. (2017). Nasopharyngeal infection by streptococcus pyogenes requires superantigen-responsive Vβ-specific T cells. Proc. Natl. Acad. Sci. USA.

[10] Miethke T., Duschek K., Wahl C., Heeg K., Wagner H. (1993). Pathogenesis of the toxic shock syndrome: T cell mediated lethal shock caused by the superantigen TSST-1. Eur. J. Immunol..

[11] Fraser J.D., Proft T. (2008). The bacterial superantigen and superantigen-like proteins. Immunol. Rev..

[12] Bohach G.A., Fast D.J., Nelson R.D., Schlievert P.M. (1990). Staphylococcal and streptococcal pyrogenic toxins involved in toxic shock syndrome and related illnesses. Crit. Rev. Microbiol..

[13] Miethke T., Wahl C., Holzmann B., Heeg K., Wagner H. (1993). Bacterial superantigens induce rapid and T cell receptor V beta-selective down-regulation of L-selectin (gp90Mel-14) in vivo. J. Immunol..

[14] Spaulding A.R., Salgado-Pabon W., Kohler P.L., Horswill A.R., Leung D.Y., Schlievert P.M. (2013). Staphylococcal and streptococcal superantigen exotoxins. Clin. Microbiol. Rev..

[15] Michie C., Scott A., Cheesbrough J., Beverley P., Pasvol G. (1994). Streptococcal toxic shock-like syndrome: Evidence of superantigen activity and its effects on T lymphocyte subsets in vivo. Clin. Exp. Immunol..

[16] Miethke T., Wahl C., Heeg K., Echtenacher B., Krammer P.H., Wagner H. (1992). T cell-mediated lethal shock triggered in mice by the superantigen staphylococcal enterotoxin b: Critical role of tumor necrosis factor. J. Exp. Med..

[17] Carlsson R., Sjogren H.O. (1985). Kinetics of IL-2 and interferon-gamma production, expression of IL-2 receptors, and cell proliferation in human mononuclear cells exposed to staphylococcal enterotoxin a. Cell. Immunol..

[18] Choi Y.W., Herman A., DiGiusto D., Wade T., Marrack P., Kappler J. (1990). Residues of the variable region of the t-cell-receptor beta-chain that interact with s. Aureus toxin superantigens. Nature.

[19] Kappler J., Kotzin B., Herron L., Gelfand E.W., Bigler R.D., Boylston A., Carrel S., Posnett D.N., Choi Y., Marrack P. (1989). V beta-specific stimulation of human T cells by staphylococcal toxins. Science.

[20] Heeg K., Gaus H., Griese D., Bendigs S., Miethke T., Wagner H. (1995). Superantigen-reactive T cells that display an anergic phenotype in vitro appear functional in vivo. Int. Immunol..

[21] Herrmann T., Baschieri S., Lees R.K., MacDonald H.R. (1992). In vivo responses of CD4+ and CD8+ cells to bacterial superantigens. Eur. J. Immunol..

[22] Huang L., Crispe I.N. (1993). Superantigen-driven peripheral deletion of T cells. Apoptosis occurs in cells that have lost the alpha/beta T cell receptor. J. Immunol..

[23] Lee W.T., Vitetta E.S. (1992). Memory T cells are anergic to the superantigen staphylococcal enterotoxin B. J. Exp. Med..

[24] MacDonald H.R., Lees R.K., Baschieri S., Herrmann T., Lussow A.R. (1993). Peripheral T-cell reactivity to bacterial superantigens in vivo: The response/anergy paradox. Immunol. Rev..

[25] Feunou P., Poulin L., Habran C., Le Moine A., Goldman M., Braun M.Y. (2003). CD4+CD25+ and CD4+CD25- T cells act respectively as inducer and effector T suppressor cells in superantigen-induced tolerance. J. Immunol..

[26] Ivars F. (2007). Superantigen-induced regulatory T cells in vivo. Chem. Immunol. Allergy.

[27] Papiernik M. (2001). Natural CD4+ CD25+ regulatory T cells. Their role in the control of superantigen responses. Immunol. Rev..

[28] Taylor A.L., Cross E.L., Llewelyn M.J. (2012). Induction of contact-dependent CD8(+) regulatory T cells through stimulation with staphylococcal and streptococcal superantigens. Immunology.

[29] Taylor A.L., Llewelyn M.J. (2010). Superantigen-induced proliferation of human CD4+CD25- T cells is followed by a switch to a functional regulatory phenotype. J. Immunol..

[30] Rink L., Nicklas W., Alvarez-Ossorio L., Fagin U., Kirchner H. (1997). Microbial superantigens stimulate T cells by the superantigen bridge and independently by a cytokine pathway. J. Interferon Cytokine Res..

[31] Mehindate K., Thibodeau J., Dohlsten M., Kalland T., Sekaly R.P., Mourad W. (1995). Cross-linking of major histocompatibility complex class II molecules by staphylococcal enterotoxin a superantigen is a requirement for inflammatory cytokine gene expression. J. Exp. Med..

[32] Palkama T., Hurme M. (1993). Signal transduction mechanisms of HLA-DR-mediated interleukin-1 beta production in human monocytes. Role of protein kinase c and tyrosine kinase activation. Hum. Immunol..

[33] Trede N.S., Castigli E., Geha R.S., Chatila T. (1993). Microbial superantigens induce NF-kappa B in the human monocytic cell line THP-1. J. Immunol..

[34] Espel E., Garcia-Sanz J.A., Aubert V., Menoud V., Sperisen P., Fernandez N., Spertini F. (1996). Transcriptional and translational control of tnf-alpha gene expression in human monocytes by major histocompatibility complex class II ligands. Eur. J. Immunol..

[35] Kim J., Urban R.G., Strominger J.L., Wiley D.C. (1994). Toxic shock syndrome toxin-1 complexed with a class II major histocompatibility molecule HLA-DR1. Science.

[36] Jardetzky T.S., Brown J.H., Gorga J.C., Stern L.J., Urban R.G., Chi Y.I., Stauffacher C., Strominger J.L., Wiley D.C. (1994). Three-dimensional structure of a human class II histocompatibility molecule complexed with superantigen. Nature.

[37] Thibodeau J., Cloutier I., Lavoie P.M., Labrecque N., Mourad W., Jardetzky T., Sekaly R.P. (1994). Subsets of HLA-DR1 molecules defined by SEB and TSST-1 binding. Science.

[38] Tiedemann R.E., Urban R.J., Strominger J.L., Fraser J.D. (1995). Isolation of HLA-DR1.(staphylococcal enterotoxin a)2 trimers in solution. Proc. Natl. Acad. Sci. USA.

[39] Sahr A., Former S., Hildebrand D., Heeg K. (2015). T-cell activation or tolerization: The yin and yang of bacterial superantigens. Front. Microbiol..

[40] Wolfle S.J., Strebovsky J., Bartz H., Sahr A., Arnold C., Kaiser C., Dalpke A.H., Heeg K. (2011). PD-L1 expression on tolerogenic apcs is controlled by STAT-3. Eur. J. Immunol..

[41] Francisco L.M., Sage P.T., Sharpe A.H. (2010). The PD-1 pathway in tolerance and autoimmunity. Immunol. Rev..

[42] Keir M.E., Butte M.J., Freeman G.J., Sharpe A.H. (2008). PD-1 and its ligands in tolerance and immunity. Annu. Rev. Immunol..

[43] Francisco L.M., Salinas V.H., Brown K.E., Vanguri V.K., Freeman G.J., Kuchroo V.K., Sharpe A.H. (2009). PD-L1 regulates the development, maintenance, and function of induced regulatory T cells. J. Exp. Med..

[44] Freeman G.J., Long A.J., Iwai Y., Bourque K., Chernova T., Nishimura H., Fitz L.J., Malenkovich N., Okazaki T., Byrne M.C. (2000). Engagement of the PD-1 immunoinhibitory receptor by a novel B7 family member leads to negative regulation of lymphocyte activation. J. Exp. Med..

[45] Riley J.L. (2009). PD-1 signaling in primary T cells. Immunol. Rev..

[46] Keir M.E., Liang S.C., Guleria I., Latchman Y.E., Qipo A., Albacker L.A., Koulmanda M., Freeman G.J., Sayegh M.H., Sharpe A.H. (2006). Tissue expression of PD-L1 mediates peripheral T cell tolerance. J. Exp. Med..

[47] Litzenburger U.M., Opitz C.A., Sahm F., Rauschenbach K.J., Trump S., Winter M., Ott M., Ochs K., Lutz C., Liu X. (2014). Constitutive ido expression in human cancer is sustained by an autocrine signaling loop involving IL-6, STAT3 and the AHR. Oncotarget.

[48] Taylor M.W., Feng G.S. (1991). Relationship between interferon-gamma, indoleamine 2,3-dioxygenase, and tryptophan catabolism. FASEB J..

[49] Munn D.H., Shafizadeh E., Attwood J.T., Bondarev I., Pashine A., Mellor A.L. (1999). Inhibition of T cell proliferation by macrophage tryptophan catabolism. J. Exp. Med..

[50] Hill M., Tanguy-Royer S., Royer P., Chauveau C., Asghar K., Tesson L., Lavainne F., Remy S., Brion R., Hubert F.X. (2007). IDO expands human CD4+CD25high regulatory T cells by promoting maturation of LPS-treated dendritic cells. Eur. J. Immunol..

[51] Mellor A.L., Munn D.H. (2004). IDO expression by dendritic cells: Tolerance and tryptophan catabolism. Nat. Rev. Immunol..

[52] Puccetti P., Grohmann U. (2007). IDO and regulatory T cells: A role for reverse signalling and non-canonical NF-kappab activation. Nat. Rev. Immunol..

[53] Terness P., Bauer T.M., Rose L., Dufter C., Watzlik A., Simon H., Opelz G. (2002). Inhibition of allogeneic T cell proliferation by indoleamine 2,3-dioxygenase-expressing dendritic cells: Mediation of suppression by tryptophan metabolites. J. Exp. Med..

[54] Giesbrecht K., Eberle M.E., Wolfle S.J., Sahin D., Sahr A., Oberhardt V., Menne Z., Bode K.A., Heeg K., Hildebrand D. (2017). IL-1beta as mediator of resolution that reprograms human peripheral monocytes toward a suppressive phenotype. Front. Immunol..

[55] Ganem M.B., De Marzi M.C., Fernandez-Lynch M.J., Jancic C., Vermeulen M., Geffner J., Mariuzza R.A., Fernandez M.M., Malchiodi E.L. (2013). Uptake and intracellular trafficking of superantigens in dendritic cells. PLoS ONE.

[56] Hopkins P.A., Fraser J.D., Pridmore A.C., Russell H.H., Read R.C., Sriskandan S. (2005). Superantigen recognition by HLA class II on monocytes up-regulates toll-like receptor 4 and enhances proinflammatory responses to endotoxin. Blood.

[57] Khan A.A., Martin S., Saha B. (2008). Seb-induced signaling in macrophages leads to biphasic TNF-alpha. J. Leukoc. Biol..

[58] Miethke T., Wahl C., Heeg K., Wagner H. (1995). Bacterial superantigens induce T cell unresponsiveness in B cell-deficient mice. Eur. J. Immunol..

[59] Wahl C., Miethke T., Heeg K., Wagner H. (1993). Clonal deletion as direct consequence of an in vivo T cell response to bacterial superantigen. Eur. J. Immunol..

[60] Van Parijs L., Ibraghimov A., Abbas A.K. (1996). The roles of costimulation and Fas in T cell apoptosis and peripheral tolerance. Immunity.

[61] Mogil R.J., Radvanyi L., Gonzalez-Quintial R., Miller R., Mills G., Theofilopoulos A.N., Green D.R. (1995). Fas (CD95) participates in peripheral T cell deletion and associated apoptosis in vivo. Int. Immunol..

[62] Strasser A., Harris A.W., Huang D.C., Krammer P.H., Cory S. (1995). Bcl-2 and Fas/APO-1 regulate distinct pathways to lymphocyte apoptosis. EMBO J..

[63] Desbarats J., Duke R.C., Newell M.K. (1998). Newly discovered role for fas ligand in the cell-cycle arrest of CD4+ T cells. Nat. Med..

[64] Hildeman D.A., Zhu Y., Mitchell T.C., Bouillet P., Strasser A., Kappler J., Marrack P. (2002). Activated T cell death in vivo mediated by proapoptotic bcl-2 family member bim. Immunity.

[65] Gonzalo J.A., Tarazona R., Schuurman H.J., Uytdehaag F., Wick G., Martinez C., Kroemer G. (1994). A single injection of staphylococcus aureus enterotoxin B reduces autoimmunity in MRL/lpr mice. Clin. Immunol. Immunopathol..

[66] Miethke T., Vabulas R., Bittlingmaier R., Heeg K., Wagner H. (1996). Mechanisms of peripheral T cell deletion: Anergized T cells are Fas resistant but undergo proliferation-associated apoptosis. Eur. J. Immunol..

[67] Scott D.E., Kisch W.J., Steinberg A.D. (1993). Studies of T cell deletion and T cell anergy following in vivo administration of SEB to normal and lupus-prone mice. J. Immunol..

[68] Chao D.T., Korsmeyer S.J. (1998). BCL-2 family: Regulators of cell death. Annu. Rev. Immunol..

[69] Newton K., Strasser A. (1998). The Bcl-2 family and cell death regulation. Curr. Opin. Genet. Dev..

[70] Mitchell T., Kappler J., Marrack P. (1999). Bystander virus infection prolongs activated T cell survival. J. Immunol..

[71] Parry R.V., Chemnitz J.M., Frauwirth K.A., Lanfranco A.R., Braunstein I., Kobayashi S.V., Linsley P.S., Thompson C.B., Riley J.L. (2005). CTLA-4 and PD-1 receptors inhibit T-cell activation by distinct mechanisms. Mol. Cell. Biol..

[72] Dagenais-Lussier X., Aounallah M., Mehraj V., El-Far M., Tremblay C., Sekaly R.P., Routy J.P., van Grevenynghe J. (2016). Kynurenine reduces memory CD4 T-cell survival by interfering with interleukin-2 signaling early during HIV-1 infection. J. Virol..

[73] Fife B.T., Bluestone J.A. (2008). Control of peripheral T-cell tolerance and autoimmunity via the CTLA-4 and PD-1 pathways. Immunol. Rev..

[74] Pierson W., Cauwe B., Policheni A., Schlenner S.M., Franckaert D., Berges J., Humblet-Baron S., Schonefeldt S., Herold M.J., Hildeman D. (2013). Antiapoptotic Mcl-1 is critical for the survival and niche-filling capacity of Foxp3(+) regulatory T cells. Nat. Immunol..

[75] Gabriel S.S., Bon N., Chen J., Wekerle T., Bushell A., Fehr T., Cippa P.E. (2016). Distinctive expression of Bcl-2 factors in regulatory T cells determines a pharmacological target to induce immunological tolerance. Front. Immunol..

